# Analysis of Gram-negative rod bacteremia in the surgical and medical ICU

**DOI:** 10.1186/cc14172

**Published:** 2015-03-16

**Authors:** D Adukauskiene, D Valanciene

**Affiliations:** 1 Lithuanian University of Health Sciences, Kaunas, Lithuania

## Introduction

The aim was to analyze the Gram-negative bacteremia profile and the predisposing factors for length of stay in the surgical and medical ICU and outcome.

## Methods

Retrospective data analysis of patients during 4 years treated in a surgical and medical ICU with positive blood culture for Gram-negative rod.

## Results

Gram-negative rod monobacteremia (*n *= 160) cultures revealed: *Escherichia coli *(*n *= 52, 32.5%), *Acinetobacter *spp. (*n *= 47, 29.4%), *Klebsiella *spp. (*n *= 22, 13.7%), *Enterobacter *spp. (*n *= 20, 12.5%), *Proteus *spp. (*n *= 11, 6.9%), anaerobes (*n *= 3, 1.9%) and other Gram-negative rods, including *Stenotrophomonas maltophilia, Haemophilus influenzae, Neisseria meningitidis, Achromobacter *spp. and *Actinobacillus limirensi *(*n *= 5, 3.1%). Both *E, coli *and *Acinetobacter *spp. were responsible for the vast majority of Gram-negative rod monobacteremia (*n *= 99, 61.8%, *P *= 0.0128). Also most often (*n *= 50, 72.5%, *P *= 0.049) primary bacteremia was caused by *E. coli *and *Acinetobacter *spp. Separate group's multidrug resistance was found: *E. coli *in 12 (23.1%) cases, *Acinetobacter *spp. in 45 (95.7%, *P *= 0.02), *Klebsiella *spp. in nine (40.9%), *Enterobacter *spp. in 11 (55.0%), *Proteus *spp. in six (54.6%) cases. The vast majority of patients with multidrug-resistant bacteremia were aged over 65 years (*n *= 64, 77.1%, *P *= 0.042), stayed in the ICU less than 14 days (*n *= 70, 84.3%, *P *= 0.039), and had lethal outcome (*n *= 74, 89.2%, *P *= 0.03). Patients who stayed in the ICU less than 14 days presented with primary Gram-negative rod bacteremia (*n *= 67, 57.7%, *P *= 0.03), need for mechanical ventilation (*n *= 90, 77.6%, *P *= 0.043) and lethal outcome (*n *= 112, 96.6%, *P *= 0.01). Lethal outcome was confirmed in patients with primary Gram-negative rod bacteremia (*n *= 55, 79.7%, *P *= 0.03), MDR strain (*n *= 74, 89.2%, *P *= 0.03), presence of shock (*n *= 120, 75.0%, *P *< 0.001), mechanical ventilation (*n *= 133, 74.3%, *P *< 0.001), cancer chemotherapy (*n *= 18, 90.0%, *P *= 0.03), and chronic obstructive pulmonary disease (*n *= 13, 100%, *P *= 0.03).

## Conclusion

*E. coli *and Acinetobacter spp. - the most often pathogens of Gram-negative rod bacteremia - were mostly multidrug resistant. Multidrug-resistant bacteremia was related to age, length of stay less than 14 days, and lethal outcome. Predisposing factors for shorter length of stay: primary bacteremia, mechanical ventilation, lethal outcome, and for lethal outcome: primary bacteremia, multidrug resistance, presence of shock, mechanical ventilation, cancer chemotherapy, chronic obstructive pulmonary disease.

